# Study on the Influence of Precipitation Characteristics on Fatigue Properties of Typical 7xxx Aluminum Alloys

**DOI:** 10.3390/ma19081601

**Published:** 2026-04-16

**Authors:** Sirui Tao, Mingyang Yu, Yanan Li, Kai Wen, Xiwu Li, Zhihui Li, Yongan Zhang, Baiqing Xiong

**Affiliations:** 1 State Key Laboratory of Nonferrous Structural Materials, China GRINM Group Co., Ltd., Beijing 100088, China; t13080992256@163.com (S.T.);; 2 GRIMAT Engineering Institute Co., Ltd., Beijing 101407, China; 3 General Research Institute for Nonferrous Metals, Beijing 100088, China

**Keywords:** 7xxx aluminum alloys, precipitation characteristics, fatigue crack growth rate prediction model

## Abstract

The mechanical response of 7xxx aluminum alloys is strongly influenced by both alloy chemistry and the resulting microstructure. In this study, the effect of precipitate characteristics on the fatigue behavior of three 7xxx aluminum alloys with different total amounts of main alloy elements was systematically investigated. Quantitative microstructural characterization was performed under T6 and T74 heat-treatment conditions by combining scanning electron microscopy, transmission electron microscopy, and electron backscatter diffraction. Meanwhile, hardness measurements, room-temperature tensile tests, and fatigue crack growth experiments were carried out to evaluate the mechanical behavior. The results show that, within the present alloy set, the over-aged condition and the alloys with higher overall alloying levels exhibited lower fatigue crack growth rates, which correlated with the coarsening of intragranular precipitates. Such microstructural evolution is suggested to facilitate dislocation motion and thereby reduce fatigue damage associated with dislocation pile-up in the present alloy set. In this work, three typical 7xxx aluminum alloys with different alloying levels were systematically investigated under T6 and T74 conditions. A statistical criterion was established to distinguish GPII zones from η′ precipitates, and a model linking precipitate characteristics to fatigue crack growth behavior was further developed. The present study aims to provide a quantitative framework for understanding and predicting the fatigue behavior of 7xxx aluminum alloys with different total amounts of main alloy elements.

## 1. Introduction

In recent years, aluminum alloys have become increasingly prevalent in manufacturing industries due to their excellent corrosion resistance, high toughness, favorable formability, and fatigue resistance [1,2]. As critical structural materials extensively used in aerospace, high-strength 7xxx aluminum alloys are pursuing higher alloying levels in response to the increasing demand for enhanced performance. Statistics show that approximately 80–90% of failures in structural materials are caused by fatigue, making fatigue performance a key indicator for assessing the safe and reliable operation of materials [3,4,5]. Consequently, improving the fatigue performance of materials and structural components has become an immediate and crucial challenge in both industrial production and practical applications.

Fatigue behavior in aluminum alloys is governed by several microstructural factors, including grain size, crystallographic orientation, intermetallic particles, and precipitate features [2,6,7,8]. A large body of recent experimental work has shown that the fatigue response of 7xxx aluminum alloys is highly sensitive to both grain size and grain orientation. The Hall–Petch [2] strengthening effect and findings by Yang et al. [9] demonstrate that grain refinement enhances the 10^7^-cycle fatigue limit of 7xxx aluminum alloys. Consistent orientation-dependent crack initiation sequences have been observed across multiple studies in 7xxx-T6/T7451 aluminum alloys, suggesting that the orientation effect is universal across aluminum alloys and particularly pronounced in high-strength 7xxx aluminum alloys [9,10,11,12,13]. Yang et al. [9] further demonstrated that in extruded 7075 bars, the <111> fiber texture localizes plastic strain in soft-oriented grains, leading to a 30% reduction in low-cycle fatigue life compared to random texture. Taken together, these studies indicate that reducing the grain size to the submicron scale or modifying grain orientation can improve the fatigue resistance of 7xxx aluminum alloys in both low-cycle and high-cycle conditions. This improvement is achieved by mitigating strain concentration, delaying crack initiation, and slowing early crack propagation under specific conditions. These findings provide definitive microstructural targets for guiding future alloy development and process optimization.

In aluminum alloys, fatigue damage is strongly affected by the size, shape, and spatial distribution of hard and brittle intermetallic particles. In 7050 and 7075 alloys, coarse particles such as Al_7_Cu_2_Fe and Al_15_Fe_4_ act as preferential sites for crack initiation, reducing fatigue life by up to 30% [10,14] and forming “fish-eye” cracks that control the fatigue limit in the very high-cycle regime [15]. Although dispersoids rich in Mn or Cr can improve the ΔK_th_ threshold, they tend to accelerate crack growth in the Paris region, resulting in a net reduction in total life [16]. Furthermore, an increased Fe content drives a shift in the crack initiation mechanism from slip bands to particle fracture, where microcrack propagation between fragments can increase the da/dN value by a factor of 2.3 [17]. Moreover, the degradation in high-cycle fatigue strength due to such coarse inclusions is irreversible, persisting even after excessive solution treatments [18].

Studies over the last several decades have identified intragranular precipitates as one of the dominant factors controlling fatigue damage in aluminum alloys. Research on 7xxx aluminum alloys indicates that these precipitates play a dominant role in governing the initiation and early propagation of fatigue cracks. For instance, retrogression and re-aging [19], which coarsens the η′ precipitates at grain boundaries while enucleating fine intragranular η′ precipitates, was shown to increase the 10^7^-cycle fatigue life of 7050 alloy by 19%. In contrast, a direct quenching process nearly doubled crack initiation life while preserving a high density of finely dispersed intragranular precipitates (4–6 nm) [20]. Gong et al. [21] further demonstrated that a peak-aged microstructure containing 3–5 nm GP and ordered η′ (MgZn_2_) precipitates can increase the 10^7^-cycle fatigue limit by approximately 20%. This improvement is attributed to the fact that the ordered precipitates promote dislocation shearing, leading to the formation of densely distributed slip bands that effectively delay strain localization. Conversely, Liu et al. [18] showed that over-aging transforms η′ precipitates into η precipitates (10–30 nm), which reduces strain intensification and widens planar slip bands to 0.2–0.3 µm, thereby making them preferred sites for fatigue crack initiation. As a result, high-cycle fatigue life decreases by 20–30%, even though the tensile strength is only slightly reduced. These findings indicate that the fatigue resistance of the alloy is highly dependent on the size, coherency, and number density of intragranular precipitates. However, previous studies have mainly provided qualitative descriptions of the microstructure–property relationship, while quantitative prediction of the fatigue behavior of 7xxx aluminum alloys remains insufficient.

To address this issue, the present study compares three typical 7xxx aluminum alloys with different total amounts of main alloy elements and associated compositional balances under T6 and T74 conditions. More specifically, it clarifies how precipitate characteristics, which are regulated by different total amounts of main alloy elements and aging treatment, affect fatigue performance. In addition, a statistically based criterion is proposed for distinguishing GPII zones from η′ precipitates, and a simplified precipitate-based framework is further used to interpret the observed fatigue crack growth behavior.

## 2. Materials and Methods

### 2.1. Materials

Three commercially manufactured domestic alloys supplied by Northeast Light Alloy Co., Ltd. (Harbin, Heilongjiang, China) with different total amounts of main alloy elements were used in this study, corresponding to Alloy-1 (12.69 wt.%), Alloy-2 (11.59 wt.%), and Alloy-3 (10.85 wt.%). The compositions of the alloys are detailed in Table 1. Each alloy was subjected to both peak-aging (T6, 120 °C/24 h) and over-aging (T74, 110 °C/8 h + 160 °C/8 h) treatments to produce distinct precipitate characteristics.

### 2.2. Microstructural Characterization

For metallographic examination, the samples were first wet-ground to remove the oxide layer and then mechanically polished to obtain a mirror-like surface. The specimens were subsequently etched with a chromic acid solution (1 mL HF + 16 mL HNO_3_ + 83 mL H_2_O + 3 g CrO_3_) (Unless otherwise specified, all chemical reagents used in this study were purchased from Beijing Tong Guang Fine Chemicals Company, Beijing, China), with the etching time adjusted according to the specimen condition. The microstructures were then examined using a Zeiss Axio Vert 200 MAT optical microscope (Carl Zeiss, Oberkochen, Germany).

Microstructural characterization by SEM was performed on a JEOL JSM-7900F microscope (JEOL Ltd., Tokyo, Japan) fitted with EBSD and Oxford EDS detectors. Before EBSD acquisition, the samples underwent mechanical polishing followed by electropolishing in a HClO_4_/C_2_H_5_OH solution with a volume ratio of 1:9. The electropolishing condition was 30 V for 5 s. EBSD data were then acquired at an accelerating voltage of 20 kV with a scanning step size of 2.0 µm.

TEM was used to identify precipitates in the aged alloys, and their size as well as number density were statistically evaluated. The morphology, spatial distribution, and crystal structure of the precipitates were determined by combining BF-TEM, SAED, and HRTEM observations. All TEM observations were performed using a Talos F200X G2 transmission electron microscope (Thermo Fisher Scientific, Eindhoven, The Netherlands) at an operating voltage of 200 kV. For TEM sample preparation, the specimens were ground to a thickness of about 50 µm, punched into circular disks with a diameter of 3 mm, and then twin-jet electropolished. The electrolyte was a mixed solution of HNO_3_ and CH_3_OH with a volume ratio of 1:3. During electropolishing, the voltage was controlled in the range of 15–20 V, the current was 60–90 mA, and the temperature was maintained between −35 and −25 °C.

The size and number density of precipitates were statistically measured with Image Pro Plus, version 6.0, using five bright-field TEM images for each sample. In addition, the thickness of the electron-transparent area in the TEM foil was determined by convergent beam electron diffraction (CBED), so as to enable calculation of the precipitate volume fraction.

### 2.3. Mechanical Properties Testing

**Figure 1:**
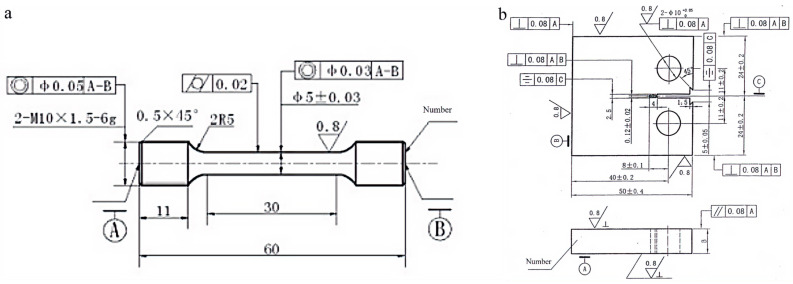
Schematic drawings of the specimens: (**a**) tensile specimen; (**b**) fatigue crack growth rate specimen.

Tensile tests at room temperature were conducted on an MTS Landmark 30 kN testing machine (MTS Systems Corporation, Eden Prairie, MN, USA) using a constant crosshead speed of 2 mm/min. All specimens were machined along the rolling direction of the plates, and the reported tensile properties for each condition were averaged over three parallel tests. The tensile specimens had a gauge length of 25 mm and a diameter of 5 mm, in accordance with the sub-size dimensions specified in ASTM E8/E8M [22], and their geometry is shown in Figure 1a. Fatigue crack growth tests were performed on an MTS-370.10 servo-hydraulic (MTS Systems Corporation, Eden Prairie, MN, USA) system according to GB/T 6398-2017 [23], using compact tension specimens with a thickness of 10 mm, as illustrated in Figure 1b. The loading orientation was L-T, and all tests were carried out under ambient conditions at a frequency of 10 Hz with a stress ratio of 0.1. The fatigue crack growth data presented here were obtained from two parallel specimens for each condition. The frequency of 10 Hz was chosen as a stable and widely used condition for room-temperature fatigue crack growth testing, ensuring adequate efficiency while minimizing possible overheating and maintaining comparability among different alloy conditions.

## 3. Results

### 3.1. Microstructure Characterization

**Figure 2:**
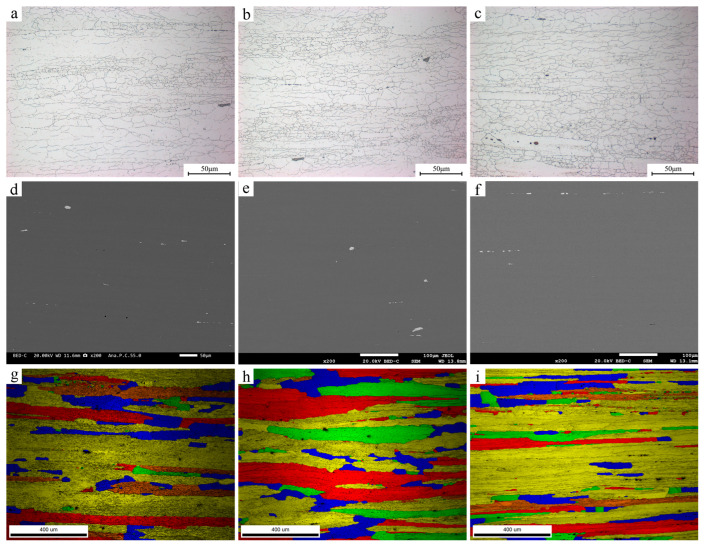
Micrographs of alloys: (**a**) Alloy-1; (**b**) Alloy-2; (**c**) Alloy-3; SEM images: (**d**) Alloy-1; (**e**) Alloy-2; (**f**) Alloy-3; EBSD misorientation images: (**g**) Alloy-1; (**h**) Alloy-2; (**i**) Alloy-3.

Figure 2 presents the OM, SEM, and EBSD results of the three investigated alloys, and the corresponding statistical data are listed in Table 2. The three alloys exhibit broadly similar grain size, grain orientation, and second-phase particle characteristics. The grain size was estimated using the manual line intercept method. Although these microstructural features are known to affect fatigue behavior, their variations among the present three alloys are relatively limited. Therefore, they were not treated in this study as the principal variables under investigation for the observed differences in fatigue crack growth behavior.

**Figure 3:**
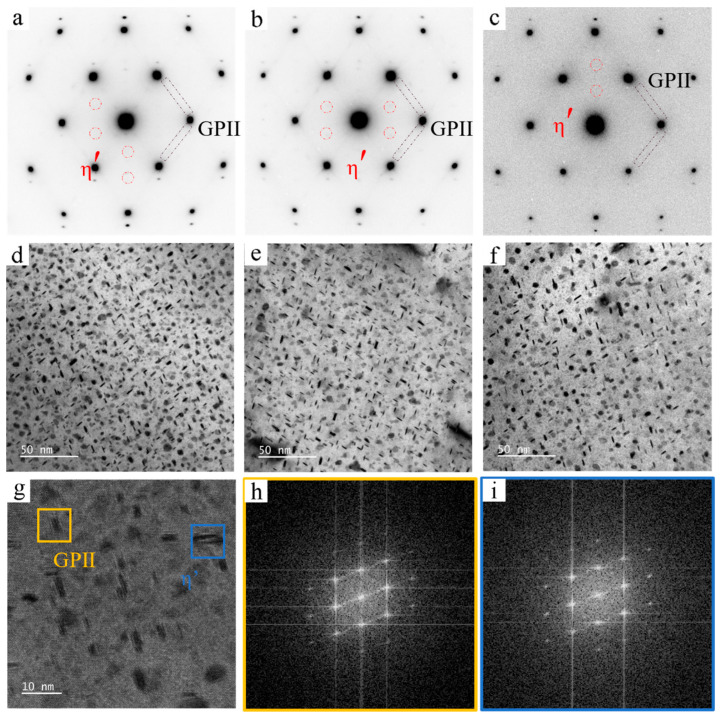
SAED patterns, bright-field (BF-TEM) images, and high-resolution (HRTEM) images along the <110>_Al_ zone axis for T6 temper alloys with different alloying levels. SAED patterns: (**a**) Alloy-1; (**b**) Alloy-2; (**c**) Alloy-3; BF-TEM images: (**d**) Alloy-1; (**e**) Alloy-2; (**f**) Alloy-3; (**g**–**i**) HRTEM images and corresponding FFT analysis for Alloy-2.

The precipitate phases commonly found in 7xxx aluminum alloys include GPI zones, GPII zones, η′ precipitates, and η precipitates, and they can be identified by SAED. Figure 3 combines the SAED patterns and TEM images of the three alloys in the T6 temper. In the T6 temper, all alloys contain a large number of fine matrix precipitates with a relatively uniform distribution. The SAED results show diffuse streaks along the ⟨111⟩_Al_ direction, which are associated with GPII zones, together with diffraction spots at the 1/3{220} and 2/3{220} positions that indicate the presence of η′ precipitates. Thus, both GPII zones and η′ precipitates are present in the three peak-aged alloys. Since GPII zones act as precursors of η′ precipitates, the two phases share a similar disk-like morphology and both preferentially precipitate on the {111}_Al_ habit planes. Figure 3g shows the HRTEM image of Alloy-2, where the yellow and blue boxes indicate the GPII zone and η′ precipitates, respectively; Figure 3h,i are the corresponding FFT patterns. The HRTEM and FFT results for the Alloy-2 sample further show that the two phases have similar thicknesses but clearly different diameters.

**Figure 4:**
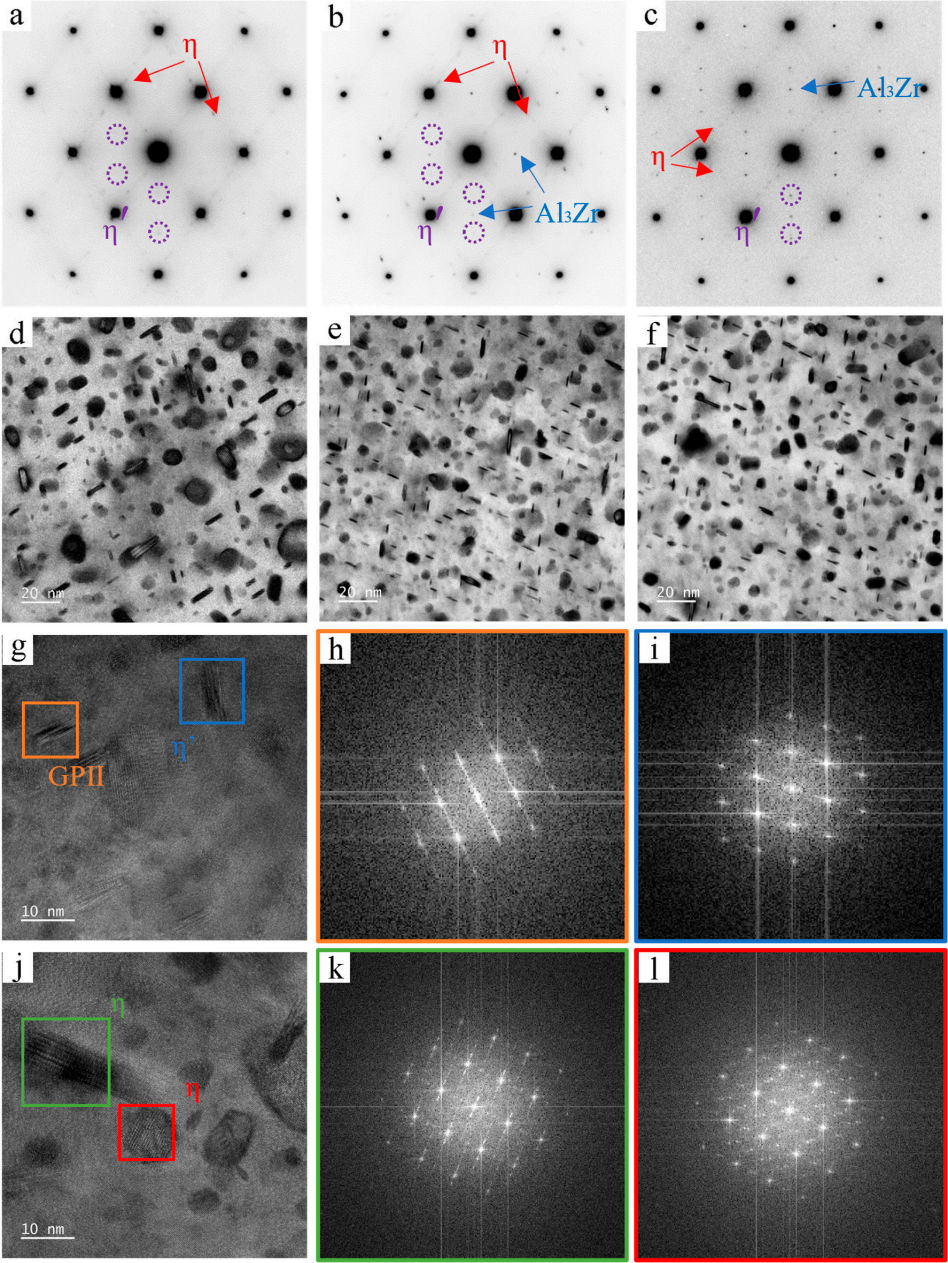
SAED patterns, bright-field (BF-TEM) images, and high-resolution (HRTEM) images along the <110>_Al_ zone axis for T74 temper alloys with different alloying levels. SAED patterns: (**a**) Alloy-1; (**b**) Alloy-2; (**c**) Alloy-3; BF-TEM images: (**d**) Alloy-1; (**e**) Alloy-2; (**f**) Alloy-3; (**g**–**l**) HRTEM images and corresponding FFT analysis for Alloy-2.

Figure 4 shows the SAED and BF-TEM results for the alloys in the T74 temper. Compared with the peak-aged condition, the precipitates in the over-aged alloys are obviously coarser. Figure 4g shows the HRTEM image of Alloy-2, where the orange and blue boxes indicate the GPII zone and η′ precipitates, respectively; Figure 4h,i are the corresponding FFT patterns. In Figure 4j, the green and red boxes indicate the η phase, and Figure 4k,l show the corresponding FFT patterns. FFT analysis of the Alloy-2 sample indicates that the finer precipitates are still GPII zones and η′ precipitates, whereas the coarse particles correspond to the η phase.

### 3.2. Mechanical Properties

**Figure 5:**
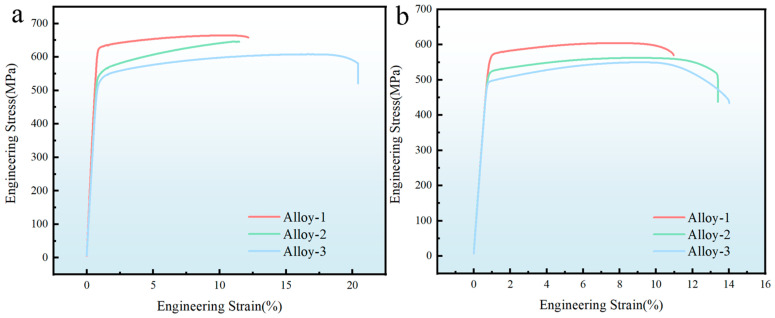
Stress–strain curves for alloys with different alloying levels: (**a**) T6 temper; (**b**) T74 temper.

Figure 5 presents the room-temperature tensile stress–strain responses of alloys with different total amounts of main alloy elements under different aging conditions. For the T6 temper, the strength increases with different total amounts of main alloy elements within the present alloy set, mainly because GPII zones and especially η′ precipitates provide stronger precipitation strengthening under T6 temper. With increasing total amount of main alloy elements, a greater number of η′ precipitates are formed, which obstruct dislocation movement through the bypass mechanism, thereby enhancing strength. Compared with the T6 state, all alloys in the T74 temper exhibit lower strength because over-aging leads to precipitate coarsening and transformation toward the more stable η phase. Despite this, Alloy-1 maintains stronger strength due to a higher volume of η precipitates, which continue to impede dislocation movement.

The crack length was evaluated from the experimental records obtained during fatigue crack growth testing on the MTS-370.10 servo-hydraulic system. The recorded data were statistically analyzed and fitted to generate the a-N and da/dN-ΔK curves. The fatigue crack growth data presented here were obtained from two parallel specimens for each condition. Given the consistent behavior between the two specimens, results from one representative specimen are shown.

**Figure 6:**
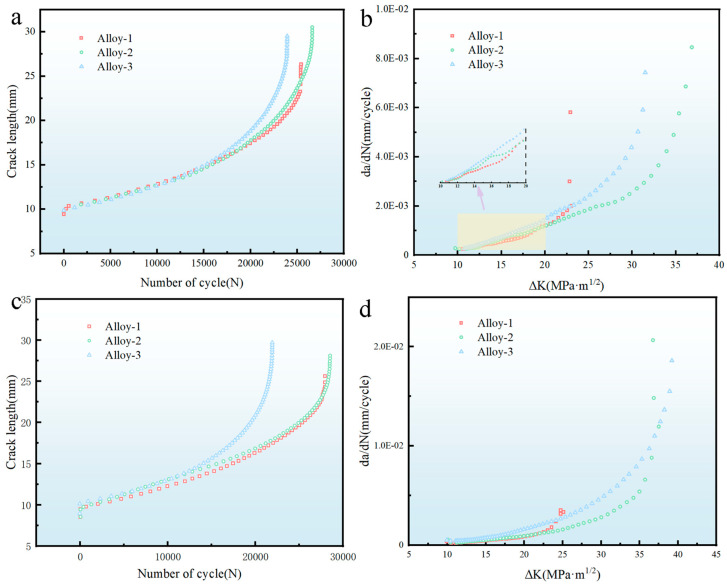
Fatigue crack growth curves of alloys with different total amounts of main alloy elements under different tempers: T6 temper: (**a**) a-N curves; (**b**) da/dN-ΔK curves; T74 temper: (**c**) a-N curves; (**d**) da/dN-ΔK curves.

Figure 6 compares the fatigue crack growth behavior of the alloys with different total amounts of main alloy elements in the T6 and T74 tempers. The a-N curves in Figure 6a,c indicate that the crack length in all three alloys increases continuously with the number of cycles. In the early stage of crack propagation (<10,000 cycles), crack extension remains relatively slow and the difference among the three alloys is not obvious. As cycling proceeds, the crack growth rate increases progressively and the separation among the three curves becomes more evident. At the same cycle number, the crack lengths are ranked as Alloy-1 > Alloy-2 > Alloy-3. In addition, the gap in crack length becomes larger with increasing cycle number, suggesting that Alloy-1 has the greatest resistance to fatigue crack growth.

The da/dN-ΔK curves in Figure 6b,d further show that Alloy-1 maintains the lowest crack propagation rate during both the initial and stable growth stages. Overall, the fatigue crack growth rate decreases in the order of Alloy-3, Alloy-2, and Alloy-1. Under the T6 condition, Alloy-1 shows the lowest da/dN value and thus the strongest resistance to crack advance. The same tendency is also observed in the T74 condition. This result is associated with the increase in precipitate size and amount observed with increasing overall total amount of main alloy elements within the present alloy set. During crack propagation, these precipitates promote more frequent crack deflection and local kinking, which strengthens the roughness-induced crack closure effect. As a result, resistance to fatigue crack growth is improved.

## 4. Discussion

### 4.1. Statistical Analysis of Disk-Shaped Precipitates

**Figure 7:**
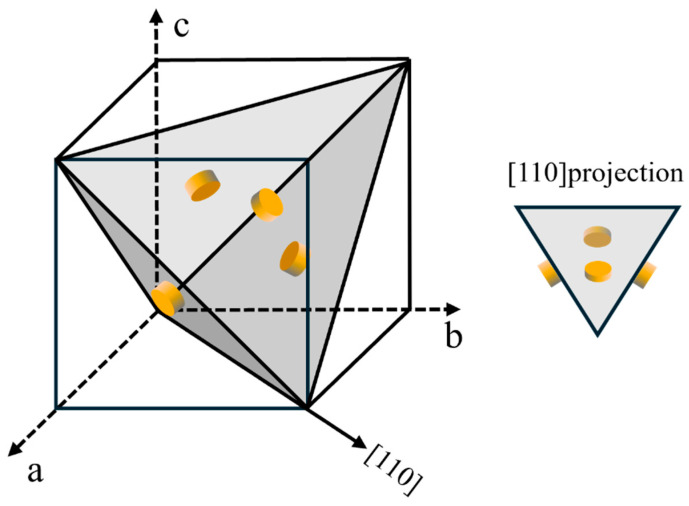
Disk-shaped phases (GPII zone and η′ precipitates) on the {111}_Al_ planes and the projected view observed in the [110]_Al_ direction.

For the alloy in the T6 temper, the matrix precipitates are mainly composed of GPII zones and η′ precipitates, and both phases display a disk-like morphology, as illustrated in Figure 7. In the peak-aged condition, these two types of disk-shaped precipitates cannot be clearly separated in conventional bright-field TEM images, whereas HRTEM observations allow reliable identification. As indicated by the preceding analysis, the thickness difference between GPII zones and η′ precipitates is very limited, while their diameters differ more obviously. This suggests that the evolution from GPII zones to η′ precipitates is associated mainly with an increase in diameter rather than with substantial thickening. In the present alloy system, a statistical diameter threshold was therefore adopted for practical phase classification.

**Figure 8:**
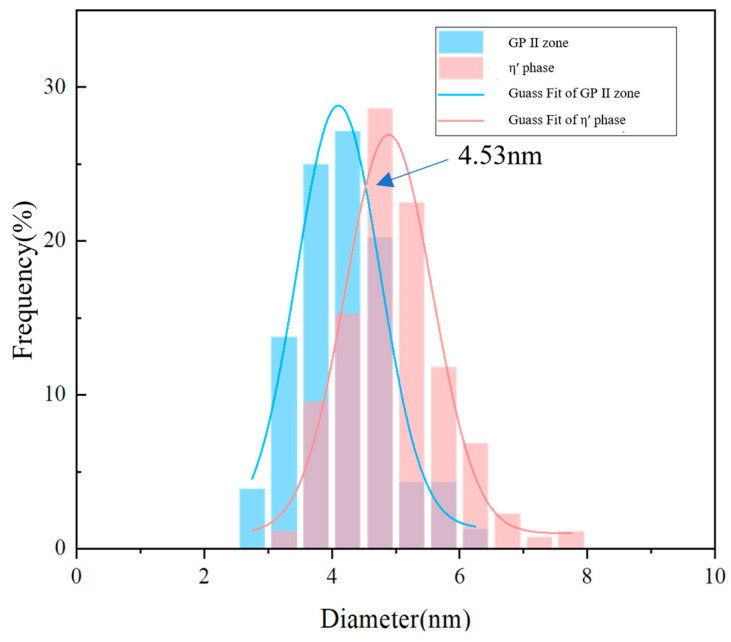
Diameter distribution histogram of GPII zones and η′ precipitates in the T6 temper alloy.

For quantitative analysis of these two phases, HRTEM images were used to identify the disk-shaped precipitates and to measure their dimensions statistically. Because the observation area in HRTEM is limited, a large number of images from different positions in all three alloys were systematically examined, including regions inside grains, as well as areas adjacent to grain boundaries with different grain orientations. For each sample, at least 80 independent HRTEM regions were analyzed. On this basis, no fewer than 600 measurements were collected for both GPII zones and η′ precipitates in every alloy, thereby ensuring acceptable statistical reliability. Although the observation area of HRTEM is inherently limited, multiple positions within grains and near grain boundaries were examined to improve representativeness as much as possible. The diameter distributions of the two precipitate populations were then fitted by Gaussian functions, and the fitted curves are presented in Figure 8. A clear bimodal distribution was obtained, and the intersection of the two fitted curves was located at 4.53 nm. The diameters of the GPII zones fall within 2.5–6 nm, and most of them are concentrated in the range of 3–4.5 nm. By comparison, the η′ precipitates are distributed between 3 and 8 nm and are mainly concentrated within 4.5–7 nm. Since these two phases cannot be reliably separated in conventional BF-TEM images, the statistically obtained diameter of 4.53 nm was used in this study as the boundary for phase classification. This treatment made it possible to distinguish the two precipitate populations quantitatively in BF-TEM observations and thus to calculate their strengthening contributions separately. In addition, fitting based on the combined size data from all three alloys produced the same threshold, whereas separate fitting for individual alloys resulted only in slight deviations within the experimental uncertainty, indicating that this classification boundary is stable for the present alloy system.

### 4.2. Determination of Precipitate Volume Fractions

**Figure 9:**
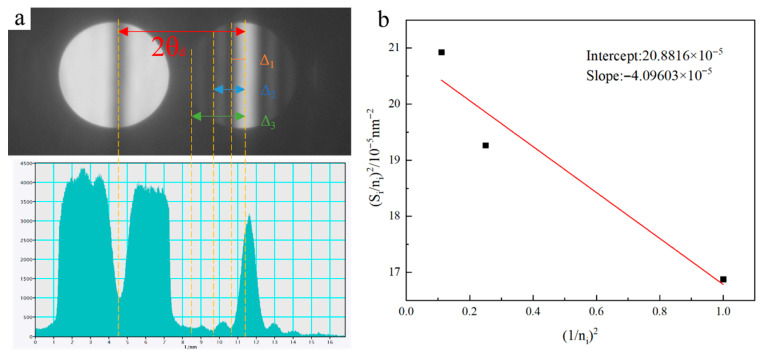
Determination of sample thickness in the TEM observation area using the CBED method: (**a**) CBED pattern under two-beam conditions; (**b**) linear fitting of (1/*n_i_*)^2^ − (*s_i_*/*n_i_*)^2^.

To further quantitatively analyze the precipitates in peak-aging alloys, the volume fraction of these phases was calculated. To obtain the volume fraction, the sample thickness within the TEM observation area required precise measurement. The sample thickness was determined using the CBED method [24], yielding the final thickness t. Figure 9 shows the calculation of the thin-area thickness for Alloy-2.

The volume of the observed sample region is given by Equation (1):

(1) $$V_{\mathrm{sample}}=t\times A$$

where A is the area of the selected observation region. The volume of an individual disk-shaped precipitate was approximated by a cylindrical model, as expressed in Equation (2):

(2) $$V_{\mathrm{precipitate}}=\pi R^{2}T$$

where R and T denote the precipitate radius and thickness. Because these precipitates form on four equivalent {111}_Al_ habit planes, the total precipitate volume can be written as Equation (3):

(3) $$V_{\mathrm{precipitate}}^{\mathrm{total}}=4N\left(111\right)\bar{V_{\mathrm{precipitate}}}=2N\left(111\right)\bar{V_{\mathrm{plate}}}$$

where N(111) is the number of precipitates on the (111)_Al_ and $\bar{V_{\mathrm{precipitate}}}$ and *V_plate_* represent the average volumes of the precipitates and the observed plate-shaped projections, respectively. The precipitate volume fraction was then calculated by Equation (4):

(4) $$f_{v}=\frac{V_{\mathrm{precipitate}}^{\mathrm{total}}}{V_{\mathrm{sample}}}$$

Although the precipitate volume fractions were calculated based on CBED thickness measurements and geometric approximations, uncertainties associated with counting statistics, local thickness variation, and stereological assumptions should be acknowledged. The volume fractions of GPII zones and η′ precipitates in the T6 temper alloys were obtained, as listed in Table 3. With increasing total amount of main alloy elements, the precipitates become larger on average, while the fraction of GPII zones decreases and that of η′ precipitates increase.

For the η precipitates in over-aged alloys, the volume of an individual precipitate was approximated from its long-axis diameter using Equation (5) [25]:

(5) $$V_{\mathrm{precipitate}}=\frac{0.38\pi D^{3}}{4}$$

The calculated volume fractions are summarized in Table 4. Compared with the T6 temper, both the number density and the fraction of GPII zones and η′ precipitates decrease markedly, while η precipitates become the dominant phase in the T74 temper alloys. As the total amount of main alloy elements increases, the proportion of η precipitates further rises, indicating enhanced precipitate coarsening in the over-aged condition.

### 4.3. Strength Model

The superior strength of 7xxx aluminum alloys is primarily derived from precipitation strengthening. In the present section, the strengthening analysis is intended to interpret the monotonic tensile/yield response of the investigated alloys. Although precipitate characteristics also influence fatigue behavior, the governing mechanisms for fatigue crack growth under cyclic loading are not identical to those for monotonic strengthening and are therefore discussed separately in Section 4.5. For these alloys, precipitation strengthening is commonly interpreted in terms of two mechanisms: dislocation shearing and Orowan bypassing [26,27,28,29].

For the shearing mode, the strengthening increment includes coherency strengthening (Δσ_cs_), modulus mismatch strengthening (Δσ_ms_) and order strengthening (Δσ_os_) [30,31]. In the present calculation, the shearing contribution was taken as the larger value between Δσ_cs_ + Δσ_ms_ and Δσ_os_ [32]. The corresponding expressions are given below.

(6) $${\Delta \;\sigma }_{cs}=M\alpha_{\varepsilon }{\left(G\varepsilon_{c}\right)}^{\frac{3}{2}}{\left(\frac{2rf}{Gb}\right)}^{\frac{1}{2}}$$

(7) $${\Delta \;\sigma }_{ms}=M0.0055{\left(\Delta G\right)}^{\frac{3}{2}}{\left(\frac{2f}{G}\right)}^{\frac{1}{2}}{\left(\frac{r}{b}\right)}^{\frac{3m}{2}-1}$$

(8) $${\Delta \;\sigma }_{os}=M0.81\frac{\gamma_{apb}}{2b}{\left(\frac{3\pi f}{8}\right)}^{\frac{1}{2}}$$

In these equations, M is the Taylor factor [29]; G is the shear modulus of the matrix [33]; α_ε_ and m are constants; and r and f denote the average radius and volume fraction of the precipitates, respectively. The phase-dependent parameters, including the constrained lattice misfit ε_c_, the modulus mismatch ΔG, and the antiphase-boundary energy γ_apb_, were selected from the reported values for GP zones, η′ precipitates, and η precipitates [33,34,35,36,37,38]. Other constants were taken according to the corresponding models and are not repeated here.

For non-shearable precipitates, the strengthening increment was evaluated using the Orowan bypass mechanism, in which dislocations loop around the particles [27]. The corresponding expression is given in Equation (9):

(9) $${\Delta \;\sigma }_{\mathrm{pass}}=M\frac{0.4Gb}{\pi \sqrt{1-\upsilon }}\frac{ln\left(2\sqrt{2/3}r/b\right)}{\lambda }$$

where υ = 0.33 is Poisson’s ratio [39]; and λ is the inter-precipitate spacing defined by Equation (10) [40]:

(10) $$\lambda =2\sqrt{2/3}r\left(\sqrt{\pi /4f}-1\right)$$

For the three alloys with different total amounts of main alloy elements, the precipitates in the peak-aged condition are composed of GPII zones and η′ precipitates, whereas the T74 temper contains these two phases together with η precipitates. As discussed above, GPII zones and η precipitates were assigned to the shearing and bypass mechanisms, respectively, while the appropriate strengthening mode for η′ precipitates still needed to be determined. When the statistical data for η′ precipitates were substituted into both the shearing model and the Orowan bypass model, the calculated results showed that, for the peak-aged alloy, the strengthening increment obtained from the bypass model was more appropriate than that from the shearing model. This suggests that η′ precipitates should be treated as bypassed precipitates in the present calculation. For GPII zones, which follow the shearing mechanism, the calculated value of Δσ_cs_ + Δσ_ms_ is markedly larger than Δσ_os_ in the peak-aged alloy, indicating that the strengthening contribution of GPII zones should be taken as the sum of coherency strengthening and modulus mismatch strengthening. The strengthening increment of the η phase, which follows the bypass mechanism, was then calculated directly from the corresponding expression.

Using the precipitate statistics and the strengthening models described above, the precipitation-strengthening increments of the peak-aged alloys with different total amounts of main alloy elements were calculated, and the results are presented in Table 5.

The calculated yield strengths of the T6 temper alloys show the same variation tendency as the experimental data. The above calculations are intended to account for the monotonic strengthening contribution of precipitates to the yield response. In all cases, the predicted values are slightly lower than the measured ones, and the deviations range from 2 to 20 MPa. The prediction error remains within 7.2%, indicating that the model provides a reasonable estimate of the yield strength.

### 4.4. Validation of the Model Fitting

To examine whether the strength model established for the peak-aged alloy can also be applied to the T74 condition, the T74 temper alloy was used for validation. The precipitation statistics listed in Table 4 were introduced into the corresponding expressions to calculate the strengthening contributions of each precipitate phase, and the results are summarized in Table 6. The comparison shows that the model remains applicable to the T74 temper alloy, which is also consistent with the practical usefulness of the previously adopted 4.53 nm statistical classification threshold for the present alloy system.

### 4.5. Fatigue Crack Growth Model

Fatigue crack growth in the Paris regime is governed by cyclic slip, crack-tip plastic deformation, and the interaction between dislocations and precipitates during repeated loading. Therefore, although precipitate characteristics affect both strength and fatigue behavior, the relevant mechanisms for fatigue crack growth must be considered separately from those controlling the monotonic tensile response.

When dislocations cut through fine and relatively dispersed precipitates, such as GPII zones and η′ precipitates, planar slip may be more readily activated during cyclic deformation, which can facilitate microcrack initiation and promote crack growth under certain conditions. By contrast, for coarser precipitates such as the η phase, dislocation bypassing becomes more likely during cyclic deformation. This may alter slip localization, crack-path tortuosity, and crack-closure behavior, thereby contributing to a lower fatigue crack growth rate in the present alloy set. On this basis, a fatigue crack growth model for 7xxx aluminum alloys was developed by correlating the relevant model parameters with precipitate characteristics.

Using the Alloy-2 material as an example, the effect of microstructure on fatigue crack growth was incorporated on the basis of the Paris equation [41]

(11) $${\frac{da}{dN}=C\left(\Delta K\right)}^{m}$$

where da/dN is the crack growth rate, ΔK is the stress intensity factor range, and C and m are constants determined by the material and loading condition.

Fatigue crack growth rate in the Paris zone is governed by the dislocation slip distance ℓ. The wider the slip zone, the greater the slip distance ℓ, the larger the tip blunting/resharpening amplitude, and the further the crack front advances by one unit. Consequently,

(12) $$\frac{da}{dN}\propto l$$

The slip distance ℓ = area swept per activation × number of successful activations per unit time; the number of successful activations is proportional to the thermal activation rate, so

(13) l∝ rate

According to the heat activation theory [42]

(14) $$\mathrm{rate}\propto \exp\left(-\frac{\Delta G}{KT}\right)$$

where ΔG is the activation enthalpy, K is the Boltzmann constant, and T is the absolute temperature. Activation enthalpy originates from the opposing resistance felt by dislocations due to line tension. Specifically, when a dislocation is anchored by a nano-precipitated phase and bent, line tension generates a restoring force directed toward the curvature center at both ends of the dislocation line. The corresponding critical shear stress is defined herein as the back-tracking stress τ_back_.

(15) $$\Delta G\propto \tau_{\mathrm{back}}$$

When a dislocation is anchored by particles spaced at L and bent to R ≈ L/2, the applied shear stress required to maintain equilibrium is

(16) $$\tau_{\mathrm{back}}=T/\left(bR\right)$$

T represents line tension [43]

(17) $$T=\alpha Gb^{2}$$

where α is the dislocation coefficient, with 1 for straight dislocations and 0.5 for bent dislocations; G is the shear modulus, which is 25 GPa for aluminum alloys; and b is the Bravais vector, which is 0.286 for FCC metals.

(18) $$\tau_{\mathrm{back}}=\alpha \mathrm{Gb}/R$$

For randomly distributed nanoparticles, the mean free path [44] is

(19) $$L\approx 1/\sqrt{f_{v}r}$$

Substituting R ≈ L/2 into Equation (16) yields

(20) $$\tau_{\mathrm{back}}\approx 2\alpha \mathrm{Gb}\sqrt{f_{v}r}$$

where f_v_ is the volume fraction of the precipitated phase and r is the average radius of the precipitated phase. It can be obtained that

(21) $$\tau_{\mathrm{back}}\propto \sqrt{f_{v}r}$$

Based on the above formulas, we can obtain

(22) $$\frac{da}{dN}\propto \exp\left(-\mathrm{const}\sqrt{f_{v}r}\right)$$

Const represents a constant, defined as θ. Therefore, the general form of the fatigue crack growth rate model derived in this paper is:

(23) $${\frac{da}{dN}=\exp\left(-\theta \sqrt{f_{v}r}\right)C\left(\Delta K\right)}^{m}$$

Substituting the relevant data for Alloy-2 in the T6/T74 temper into Equation (23) yields a constant θ value of 0.20. Therefore, the equation can be simplified to:

(24) $${\frac{da}{dN}=\exp\left(-0.20\sqrt{f_{v}r}\right)C\left(\Delta K\right)}^{m}$$

where f_v_ is the volume fraction of the precipitate phase and r is the average radius of the precipitate phase.

### 4.6. Validation of the Fatigue Crack Growth Model

To validate the applicability of the proposed fatigue crack growth model, model validation was conducted using the Alloy-1 and Alloy-3 conditions under both T6 and T74 tempers. The statistics for the volume fraction and average size of the precipitate phases are listed in Table 3 and Table 4. Substituting these values into the model yields the calculated results summarized in Table 7. The calculated values show reasonable agreement with the experimental data within the present alloy set.

## 5. Conclusions

(1) In this study, a statistical criterion was established to distinguish GPII zones from η′ precipitates. Based on the size-distribution analysis and Gaussian fitting, the diameter threshold used for phase classification was determined to be 4.53 nm.
(2) As the aging condition changed from T6 to T74 and as the total amount of main alloy elements increased, the precipitate population evolved toward a larger characteristic size and altered phase fractions. This microstructural evolution was associated with improved resistance to fatigue crack growth, i.e., a gradual decrease in the fatigue crack growth rate.
(3) Within the present alloy set, alloys with a higher total amount of main alloy elements exhibited a precipitate population with a larger characteristic size and changed distribution features, which was accompanied by a lower fatigue crack growth rate. This effect should be interpreted in terms of cyclic deformation and crack-propagation behavior, rather than as a simple extension of monotonic strengthening.

## Figures and Tables

**Table 1:** Chemical compositions of the investigated alloys (wt.%).

-	Mg	Cu	Zn	Si	Mn	Cr	Fe	Zr	Ti
Alloy-1	2.09	1.64	8.96	0.0098	<0.01	<0.01	0.022	0.10	0.021
Alloy-2	1.90	1.77	7.92	0.012	<0.01	<0.01	0.032	0.11	0.025
Alloy-3	2.24	2.29	6.32	0.010	<0.01	<0.01	0.026	0.10	0.022

**Table 2:** Statistical results of microstructure.

Alloy	Second Phase Size/μm	Second Phase Area Fraction/%	Grain Size/μm	Grain Average Misorientation < 1/%
Alloy-1	3.65	0.153	4.87	25.3
Alloy-2	3.68	0.144	4.71	27.1
Alloy-3	3.72	0.131	4.83	27.8

**Table 3:** Calculated volume fractions of precipitates in T6 temper alloys (%).

Alloy	GPII Zone	η′ Precipitates
Alloy-1	0.157	0.301
Alloy-2	0.185	0.258
Alloy-3	0.205	0.249

**Table 4:** Calculated volume fractions of precipitates in T74 temper alloys (%).

Alloy	GPII Zone	η′ Precipitates	η Precipitates
Alloy-1	0.0082	0.2411	1.6935
Alloy-2	0.0173	0.3243	1.5131
Alloy-3	0.0206	0.4839	0.8677

**Table 5:** The comparison between the calculated values from classical strengthening models and experimental values in T6 temper alloys.

Alloy	Δ*σ_cs_*/MPa	Δ*σ_ms_*/MPa	Δ*σ_pass_*/MPa	*σ_p_*/MPa	Experiment/MPa
Alloy-1	172.129	0.002	69.010	241.141	260
Alloy-2	317.125	0.003	84.179	401.307	399
Alloy-3	186.541	0.002	61.551	248.094	230

**Table 6:** The comparison between the calculated values from strengthening models and experimental values in T74 temper alloys.

Alloy	Δ*σ_shear_*/MPa	Δ*σ_pass_*/MPa	*σ_p_*/MPa	Experiment/MPa
Alloy-1	38.6	176.6	215.2	226
Alloy-2	83.2	266.8	350	344
Alloy-3	23.1	128.9	163	152

**Table 7:** The comparison between the calculated values from FCG models and experimental values in alloys.

Alloy	Temper	Calculated Values		Experimental Values	
		ΔK = 12 MPa·m^1/2^	ΔK = 15 MPa·m^1/2^	ΔK = 12 MPa·m^1/2^	ΔK = 15 MPa·m^1/2^
Alloy-2	T6	3.42 × 10^−4^	6.99 × 10^−4^	3.49 × 10^−4^	7.13 × 10^−4^
	T74	2.36 × 10^−4^	3.87 × 10^−4^	2.43 × 10^−4^	3.98 × 10^−4^
Alloy-3	T6	3.51 × 10^−4^	7.09 × 10^−4^	3.58 × 10^−4^	7.18 × 10^−4^
	T74	2.59 × 10^−4^	4.36 × 10^−4^	2.68 × 10^−4^	4.43 × 10^−4^

## Data Availability

The original contributions presented in this study are included in the article. Further inquiries can be directed to the corresponding authors.
